# Volumetric and diffusion MRI longitudinal patterns in brain metastases after laser interstitial thermal therapy

**DOI:** 10.1007/s00330-025-11587-0

**Published:** 2025-04-18

**Authors:** Francesco Sanvito, Jingwen Yao, Gianluca Nocera, Guowen Shao, Zexi Wang, Nicholas S. Cho, Ashley Teraishi, Catalina Raymond, Kunal Patel, Nader Pouratian, Richard G. Everson, Isaac Yang, Noriko Salamon, Won Kim, Benjamin M. Ellingson

**Affiliations:** 1 UCLA Brain Tumor Imaging Laboratory (BTIL), Center for Computer Vision and Imaging Biomarkers, University of California Los Angeles, Los Angeles, CA USA; 2 Department of Radiological Sciences, David Geffen School of Medicine, University of California Los Angeles, Los Angeles, CA USA; 3 Università Vita-Salute San Raffaele, Milano, Italy; 4 Neuroradiology Unit and CERMAC, IRCCS Ospedale San Raffaele, Milano, Italy; 5 Department of Neurosurgery and Gamma Knife Radiosurgery, IRCCS Ospedale San Raffaele, Milan, Italy; 6 Department of Bioengineering, Henry Samueli School of Engineering and Applied Science, University of California Los Angeles, Los Angeles, CA USA; 7 Medical Scientist Training Program, David Geffen School of Medicine, University of California Los Angeles, Los Angeles, CA USA; 8 David Geffen School of Medicine, University of California Los Angeles, Los Angeles, CA USA; 9 Department of Neurosurgery, Ronald Reagan UCLA Medical Center, University of California Los Angeles, Los Angeles, CA USA; 10 Department of Neurological Surgery, UT Southwestern Medical Center, Dallas, TX USA; 11 Department of Radiation Oncology, Ronald Reagan UCLA Medical Center, University of California Los Angeles, Los Angeles, CA USA; 12 Jonsson Comprehensive Cancer Center, Ronald Reagan UCLA Medical Center, University of California Los Angeles, Los Angeles, CA USA; 13 Lundquist Institute for Biomedical Innovation at Harbor-UCLA Medical Center, Torrance, CA USA; 14 Department of Head and Neck Surgery, Ronald Reagan UCLA Medical Center, University of California Los Angeles, Los Angeles, CA USA; 15 Department of Psychiatry and Biobehavioral Sciences, David Geffen School of Medicine, University of California Los Angeles, Los Angeles, Los Angeles, CA USA

**Keywords:** Brain, Neoplasm metastasis, Magnetic resonance imaging, Diffusion magnetic resonance imaging, Laser therapy

## Abstract

**Objective:**

To characterize MRI changes of brain metastases (BM) following laser interstitial thermal therapy (LITT), particularly in lesions exhibiting durable response or early progression.

**Materials and methods:**

Longitudinal scans from patients with LITT-treated BM were retrospectively analyzed. Treatment response was categorized as durable response, long-term disease control (i.e., stable at 1 year), stable disease < 1 year, or progression < 1 year. Volumetric and diffusion MRI changes after LITT were analyzed for each subregion (contrast-enhancing, central non-enhancing, whole lesion). Volumetric changes were modeled with bi-exponential fits in responding lesions and progressors.

**Results:**

295 MRI scans from 47 lesions across 42 patients (57.8 ± 14.3 years old, males:females 21:21) were analyzed. Overall, the post-LITT scan showed a lesion enlargement (*p* < 0.0001 for all subregions), more pronounced in the contrast-enhancing (CE) component (median = +77%, *p* < 0.0001), and a reduction in the apparent diffusion coefficient (ADC) (*p* < 0.001), especially in the central non-CE component (median = −224 × 10^−^^6^ mm^2^/s, *p* < 0.0001), with no significant differences between responders and progressors. Based on mathematical modeling, the responding lesions shrank to half of the post-LITT size after 79.83 days (median “pseudo-half-life”), and the progressing lesions shrank for a median of 27 days (median time-to-growth) before regrowing. The estimated optimal timepoints for follow-up scans were 23 days and 125 days, yielding accuracy/specificity/sensitivity 0.82/1.0/0.55 in identifying progressing lesions (*p* < 0.01).

**Conclusion:**

BM typically exhibit an early volume increase with diffusion restriction after LITT. Responders then show bi-exponential shrinkage with gradual diffusion increase. Progression can usually be detected only after 3–4 months, because earlier radiographic patterns may overlap with responding lesions.

**Key Points:**

***Question*** *Laser interstitial thermal therapy (LITT) is an emerging local treatment for brain metastases, but the radiographic patterns following this treatment have not been thoroughly described.*

***Findings*** *Responding lesions showed a typical radiographic pattern with early volumetric enlargement and diffusion restriction (not exclusive of responders), followed by a bi-exponential shrinkage and diffusion elevation.*

***Clinical relevance*** *Being aware of the typical radiographic changes in brain metastases responding to LITT is informative for the interpretation of follow-up images. Early volumetric and diffusion changes (< 3–4 months) do not appear to be reliable markers to predict treatment success.*

**Graphical Abstract:**

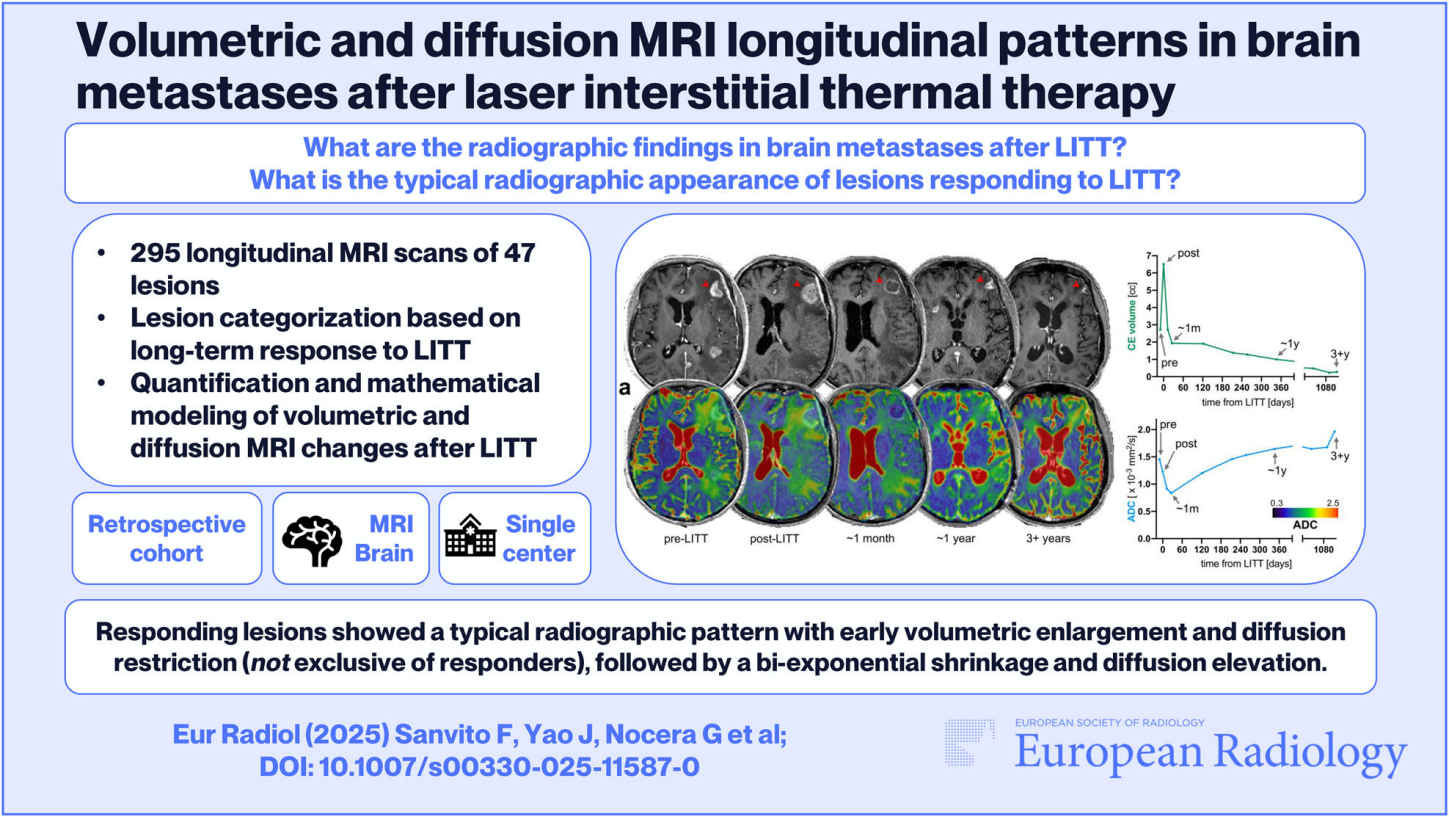

## Introduction

Laser interstitial thermal therapy (LITT) is a novel local treatment option for brain metastases (BM) [1–4]. In this procedure, a laser fiber is placed into the targeted lesion through a burr hole in the skull, and laser-induced tissue damage and/or necrosis is achieved [5–9] while the regional temperature changes are monitored in real time using MR thermometry [10]. While the first-line local treatment of BM is still based on stereotactic radiosurgery or surgical resection [1], LITT is becoming a valuable option mainly for recurrent lesions [3, 11–15]. Indeed, LITT offers a minimally invasive alternative to surgery [16] and is effective on both recurrent BM and radiation necrosis (RN) [17–19], while allowing biopsy sampling for the histopathological diagnosis of recurrent BM vs RN.

Neuroradiologists are progressively becoming more experienced in monitoring brain lesions after LITT, but the knowledge about imaging features of BM receiving LITT is still sparse and anecdotal. Indeed, the available literature is limited to historical articles describing general signal changes [20–22] and to the well-established lesion enlargement that is typically observed immediately after the procedure [2, 4, 23–25]. Rigorous studies reporting on volumetric and diffusion imaging changes are lacking, and a more comprehensive characterization of radiographic changes following LITT is yet to be achieved.

In this article, we report the volumetric and diffusion changes in BM after LITT, with a particular focus on describing the radiographic patterns of response in lesions for which treatment response or disease control is achieved. Additionally, we aim to model the volumetric changes of responding and progressing lesions, to characterize the expected volumetric trajectory over time for these two groups of lesions.

## Materials and methods

### Patient selection

For this study, additional analyses were performed on a cohort reported in a previous study with substantially different aims and methodology [2]. This cohort included patients diagnosed with BM and treated with LITT at our institution between June 2014 and August 2021. None of these lesions received consolidation stereotactic radiosurgery (SRS) after LITT. All patients provided written informed consent to have their datasets used for research purposes, as approved by the institutional IRB (IRB-21-1070). The following inclusion criteria were applied: BM diagnosis; LITT performed either on a newly diagnosed BM or on a lesion suspicious for recurrent BM or RN; availability of pre-LITT, post-LITT, and follow-up MRI.

### Image acquisition and processing

Pre-LITT and follow-up images were obtained with a head coil at either 1.5 or 3.0 T, while immediate post-LITT images were acquired at 1.5 T immediately after LITT using the same surface coil used for the MR thermometry monitoring. The MRI protocol included post-contrast T_1_-weighted images (T_1_-post) and diffusion-weighted imaging (DWI, with *b*-values 0 and 1000 s/mm^2^). The apparent diffusion coefficient (ADC) maps were automatically calculated on the scanner from DWI images, and absolute ADC values [mm^2^/s] were used for the analyses. ADC images were skull-stripped and registered to T_1_-post using *bet* and *flirt* (FSL; University of Oxford; https://fsl.fmrib.ox.ac.uk/fsl/). T_1_-post was intensity-normalized so that voxel intensity ranged from 0 to 1 (Supplementary Fig. 1), then 3D segmentations of the contrast-enhancing (CE) lesion component were obtained with a semi-manual approach, by contouring the lesion area and then keeping voxels surviving a threshold of 0.7 in the normalized T_1_-post (Supplementary Fig. 1). An additional “whole lesion” segmentation was obtained with a concatenation of built-in *fslmaths* functions from FSL *(-dilM*, *-fillh*, *-ero*) to perform a “closing” operation, followed by manual adjustments (Supplementary Fig. 1). Finally, a segmentation of the central non-CE component (e.g., central cystic or necrotic area, if present) was obtained by subtracting the CE segmentation from the whole lesion segmentation (Supplementary Fig. 1). None of these segmentations included the perilesional T_2_-hyperintense edema. A neuroradiologist with 8 years of expertise in neuroimaging (F.S.) quality-checked and refined all image registrations and tumor segmentations. An estimate of whether the LITT ablation was “supratotal” or “non-supratotal” (extent of ablation) was obtained using a methodology based on post-LITT to pre-LITT subtraction maps, described in detail in previous studies [2, 3].

### Lesion categorization

Scans were analyzed until disease progression or censoring, and lesions were categorized based on their response to the LITT treatment, employing the volumetric thresholds from mRANO [26] and RANO (response assessment in neuro-oncology) 2.0 [27, 28], as in previous LITT studies [2, 4]. Since a post-LITT lesion enlargement is expected and does not represent disease progression [2, 4, 25], progressive disease (PD) was defined as a ≥ 40% increase in CE volume compared to the post-LITT volume or compared to the after-LITT nadir volume (the “after-LITT nadir” is the smallest measured volume after LITT), and followed by either a clinical decision to re-treat the lesion or an additional ≥ 40% increase after ≥ 4 weeks. In particular, we focused on lesions progressing within the first year of follow-up (PD < 1 y). Only if a lesion did not meet criteria for PD < 1 y, a ≥ 65% decrease in CE volume compared to the pre-LITT volume, maintained in at least two scans and for ≥ 4 weeks, was categorized as durable partial response (PR). The category long-term disease control (LTDC) was assigned to lesions not meeting the criteria for durable PR, but showing stable disease at 1 year (including cases with PD after 1 year). Finally, stable disease within the first year (SD < 1 y) was assigned to lesions lost to follow-up within the first year and not meeting criteria for PR or PD. Each lesion was therefore categorized as either durable PR, PD < 1 y, LTDC, or SD < 1 y (Supplementary Fig. 2). All groups were analyzed to describe pre- vs post-LITT radiographic changes, while only durable PR vs PD < 1 y were compared in the mathematical modeling of volumetric changes, and only PR + LTDC vs PD < 1 y were compared in the mathematical modeling of diffusion changes (see the paragraph “Mathematical modeling of radiographic changes”).

### Statistical analyses

Non-parametric tests (with significance set to *p*-value < 0.05) were used because not all observations passed a Shapiro–Wilk normality test: two-sample Wilcoxon signed-rank tests (to compare paired observations), one-sample Wilcoxon signed-rank tests (to compare observations to a theoretical value), Mann–Whitney *U* tests (to compare different groups), Fisher’s exact test (to test the associations between categorical variables), Spearman coefficients (ρ, to evaluate the association between continuous variables).

### Mathematical modeling of radiographic changes

Volumetric changes of the CE component over time were modeled with bi-exponential fits at the single lesion level, starting from the post-LITT timepoint (i.e., time = 0 days corresponds to the immediate post-LITT scan on LITT day), using the *curve fit* function from *Python Scipy*. Only lesions with sufficient timepoints to allow the model fit (PD < 1 y: 3 timepoints; PR: 4 timepoints) were included.

The well-known post-LITT CE volume enlargement is reportedly due to a transient increased permeability of the blood-brain barrier (BBB) [4, 25, 29]. Therefore, we hypothesized that in PD < 1 y lesions, the CE volume after LITT can be modeled with a bi-exponential function that combines an exponential decay related to the resolution of the transitory BBB opening and an exponential growth reflecting the tumor regrowth:

1 $${{\rm{Volume}}}\left(t\right)={\beta }_{0}\cdot [\alpha \cdot {e}^{-{\beta }_{1}t}+(1-\alpha )\cdot {e}^{{\beta }_{2}t}]$$

“Volume” is the CE volume as a percentage of the post-LITT timepoint (i.e., post-LITT timepoint = 100%). “β_0_” is the y-intercept (forced to 100%). “β_1_” (exponential decay coefficient) and “β_2_” (exponential growth coefficient) were forced to a 0–0.1 range because values > 0.1 would correspond to a half-life or doubling time (respectively) of < 7 days, which can be considered biologically unlikely. Finally, “α” represents the relative weight of the two exponential components.

Similarly, we hypothesized that in PR lesions the shrinkage of CE volumes after LITT can be modeled with a bi-exponential function that combines a steeper exponential decay related to the resolution of the transitory BBB opening and another milder exponential decay reflecting the shrinkage due to treatment response:

2 $${{\rm{Volume}}}\left(t\right)={plateau}+({\beta }_{0}-{plateau})\cdot [\alpha \cdot {e}^{-{\beta }_{1}t}+(1-\alpha )\cdot {e}^{{-\beta }_{2}t}]$$

The structure of Eq. 2 is essentially similar to Eq. 1, but the sign applied to “β_2_” is negative to code for an exponential decay (instead of a growth), and the code was written to force β_1_ ≥ β_2_. Additionally, a “plateau” term is introduced, to account for potential residual CE areas (e.g., scar tissue). When time = 0 days, this equation simplifies to “plateau + (β_0_ – plateau)” (the y-intercept is again forced to β_0_ = 100%). While LTDC cases can be considered as a LITT successful outcome from a clinical perspective, they did not necessarily shrink in the long-term follow-up and therefore were not included in the bi-exponential modeling.

The modeled time-volume curves allowed to identify the earliest pair of theoretical follow-up timepoints with accuracy ≥ 0.8 in distinguishing PD < 1 y cases from PR cases while calculating a *p*-value with a Fisher’s exact test. This analysis was aimed at obtaining the recommended follow-up scan dates after LITT, using a data-driven approach (see Supplementary Methods for more detail).

Finally, the ADC changes over time (ΔADC = ADC_timepoint_− ADC_pre-LITT_), extracted from the whole lesion segmentation, were modeled using a cubic polynomial fit, starting from the pre-LITT timepoint. Unlike the volumetric modeling, the ADC modeling was performed on the entire group (not at the single lesion level) and it was only aimed to obtain an overall visualization of the longitudinal trends.

## Results

### Lesions and patients’ characteristics

A total of 295 MRI scans from 47 lesions across 42 patients (57.8 ± 14.3 years old, males:females 21:21) were analyzed (Table 1). Most lesions were recurrent and previously received SRS alone and/or surgery plus radiation therapy (RT). Approximately two-thirds of the lesions were symptomatic at the time of LITT. The majority of the lesions was supratentorial, and frontal, parietal, and white matter localizations were more common. Lung was the most common primary tumor, followed by melanoma and breast. Using post-LITT to pre-LITT subtraction maps [2, 3], the ablation was estimated as “supratotal” in 15 lesions. 33 lesions were receiving active systemic treatment at the time of LITT, 11 were off systemic treatment, and for 3 lesions no information about systemic treatments was available. For lesions achieving durable PR or LTDC, a mean of ~ 9 MRI scans per lesion, covering a mean of ~ 3 years of follow-up, were available. From a technical standpoint, the vast majority (92%) of T_1_-post images at all timepoints had high-resolution (≤ 1 × ≤ 1 × ≤ 1 mm) voxel size and isotropic geometry (Supplementary Table 1), compliant with standardized brain tumor imaging protocols (BTIPs) [30, 31]. As for DWI, datasets were acquired with heterogeneous protocols across subjects and timepoints (Supplementary Table 2). The protocol of the immediate post-LITT scan was the most homogeneous across patients, as it was consistently acquired with a surface coil at 1.5 T and with almost identical technical parameters (Supplementary Tables 1, 2).

**Table 1:** Lesion characteristics

Variable	Lesion count (%) or mean ± SD
Primary tumor	
Lung	21 (44.7)
Melanoma	8 (17.0)
Breast	7 (14.9)
Kidney/urinary tract	5 (10.6)
Other	6 (12.8)
Symptoms at time of LITT	
Symptomatic	29 (61.7)
Asymptomatic	18 (38.3)
Prior local treatments	
Treatment-naïve	3 (6.4)
S/P surgery	10 (21.3)
S/P radiation	43 (91.5)
Lesion location	
Frontal	10 (21.3)
Parietal	10 (21.3)
Other lobe	10 (21.3)
White matter	11 (23.4)
Deep gray matter	3 (6.4)
Infratentorial	3 (6.4)
Pre-LITT CE volume (cc)	2.7 ± 2.0
Estimated extent of ablation	
Supratotal	15 (31.9)
Non-supratotal	32 (68.1)
Systemic treatment at time of LITT	
Receiving treatment	33 (70.2)
Off treatment	11 (23.4)
Unknown	3 (6.4)
MRI Follow-up duration (months)	
PR category (*n* = 17)	33.1 ± 15.8
PD < 1 y category (*n* = 12)	4.1 ± 2.7
LTDC category (*n* = 4)	32.2 ± 16.7
SD < 1 y category (*n* = 14)	2.8 ± 2.5
Number of MRI scans per lesion	
PR category (*n* = 17)	9.5 ± 3.7
PD < 1 y category (*n* = 12)	4.1 ± 0.5
LTDC category (*n* = 4)	8.3 ± 0.5
SD < 1 y category (*n* = 14)	3.7 ± 0.8

*cc* cubic centimeters, *CE* contrast-enhancing, *LTDC* long-term disease control, *PD < 1 y* progressive disease within 1 year after LITT, *PR* durable response, *SD < 1 y* stable disease censored before 1 year after LITT, *S/P* status post

### Volumetric changes following LITT

**Fig. 1:**
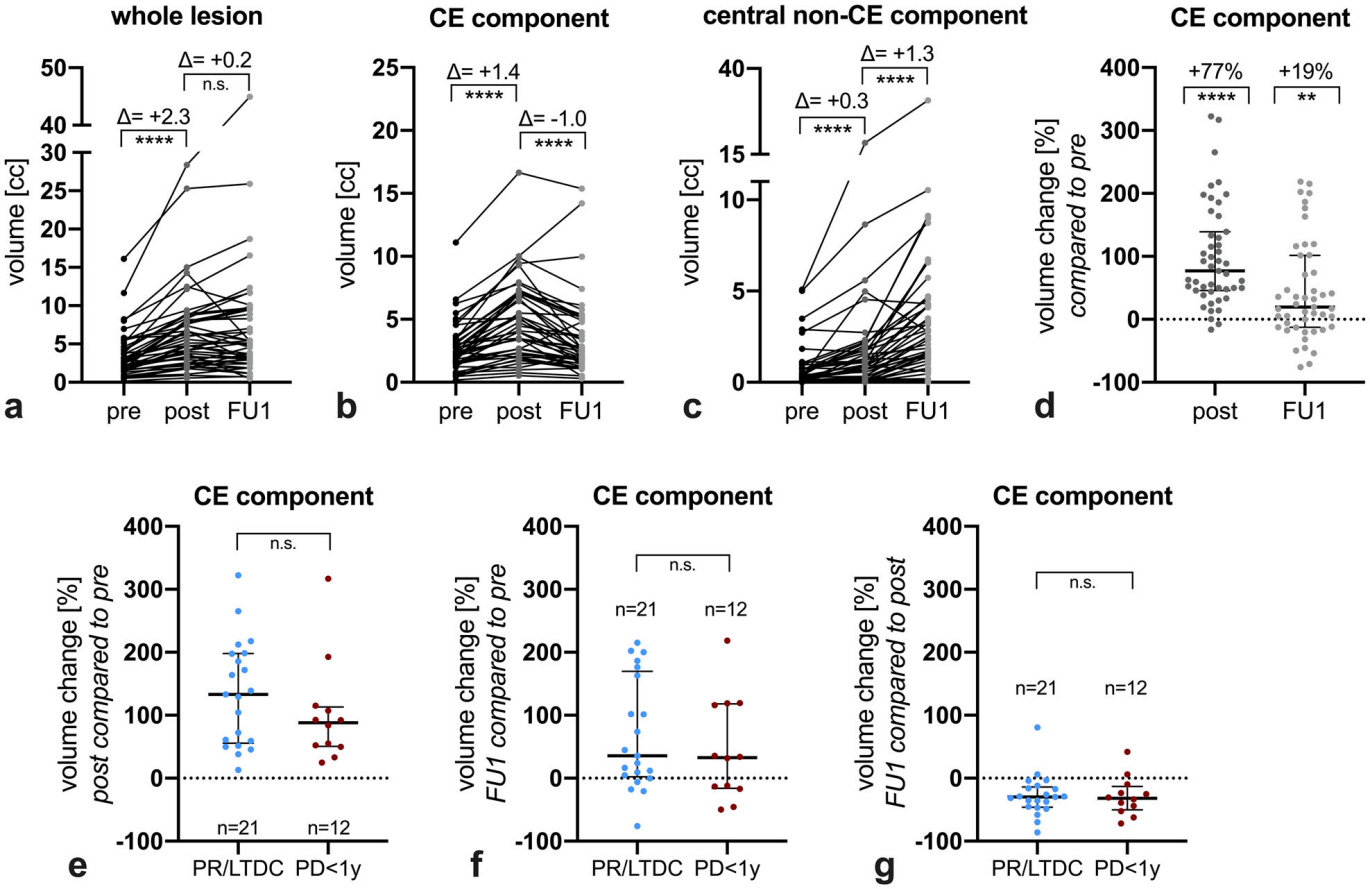
Volumetric changes following LITT. The whole lesion grows immediately after LITT and then appears substantially stable at the following scan (**a**). The CE component grows immediately after LITT and then tends to shrink at the following scan (**b**, **d**). Conversely, the central non-CE component exhibits a modest enlargement immediately after LITT and then a more pronounced enlargement at the following scan (**c**). The percent change of CE volumes at such timepoints is substantially similar between responding and progressing lesions (**e**–**g**). cc, cubic centimeters; CE, contrast-enhancing; FU1, first follow-up scan; LTDC, long-term disease control; pre, pre-LITT scan; post, immediate post-LITT scan; PD < 1 y, progressive disease within 1 year after LITT; PR, durable response

The LITT procedure determined a volumetric increase of the whole lesion (+2.3 cc median difference, *p* < 0.0001, Fig. 1a), more prominent for the CE component (+1.4 cc median difference, corresponding to +77%, *p* < 0.0001, Fig. 1b, d) and less pronounced in the central non-CE component (+0.3 cc median difference, *p* < 0.0001, Fig. 1c). On the first follow-up scan after the post-LITT MRI (FU1, acquired on a median of 33 days after LITT, IQR: 25–55 days), the whole lesion volume was overall comparable to the post-LITT volume overall (Fig. 1a) due to a combined shrinkage of the CE component (–1.0 cc median difference, *p* < 0.0001, Fig. 1b) and further enlargement of the central non-CE component (+1.3 cc median difference, *p* < 0.0001, Fig. 1c). On FU1, while smaller than the post-LITT timepoint, the CE component was still significantly larger than the pre-LITT timepoint in most lesions (+19% median, *p* < 0.01, Fig. 1d). Volume percent change on the post-LITT and FU1 timepoints did not significantly differ between treatment response categories (PR/LTDC vs PD < 1 y, Fig. 1e–g). However, data visualization allows to capture a trend toward lesions showing a post-LITT CE volume enlargement > 100% preferentially belonging to the PR/LTDC rather than to the PD < 1 y group. Post-LITT CE volume enlargement was not significantly impacted by active systemic treatments (Supplementary Fig. 3). Lesions with an estimated “supratotal” extent of ablation had a more pronounced post-LITT CE volume enlargement (Supplementary Fig. 3), and supratotal ablations showed a higher prevalence in responders, but without a statistically significant association with treatment outcomes (Supplementary Fig. 4).

### Mathematical modeling of volumetric changes

**Fig. 2:**
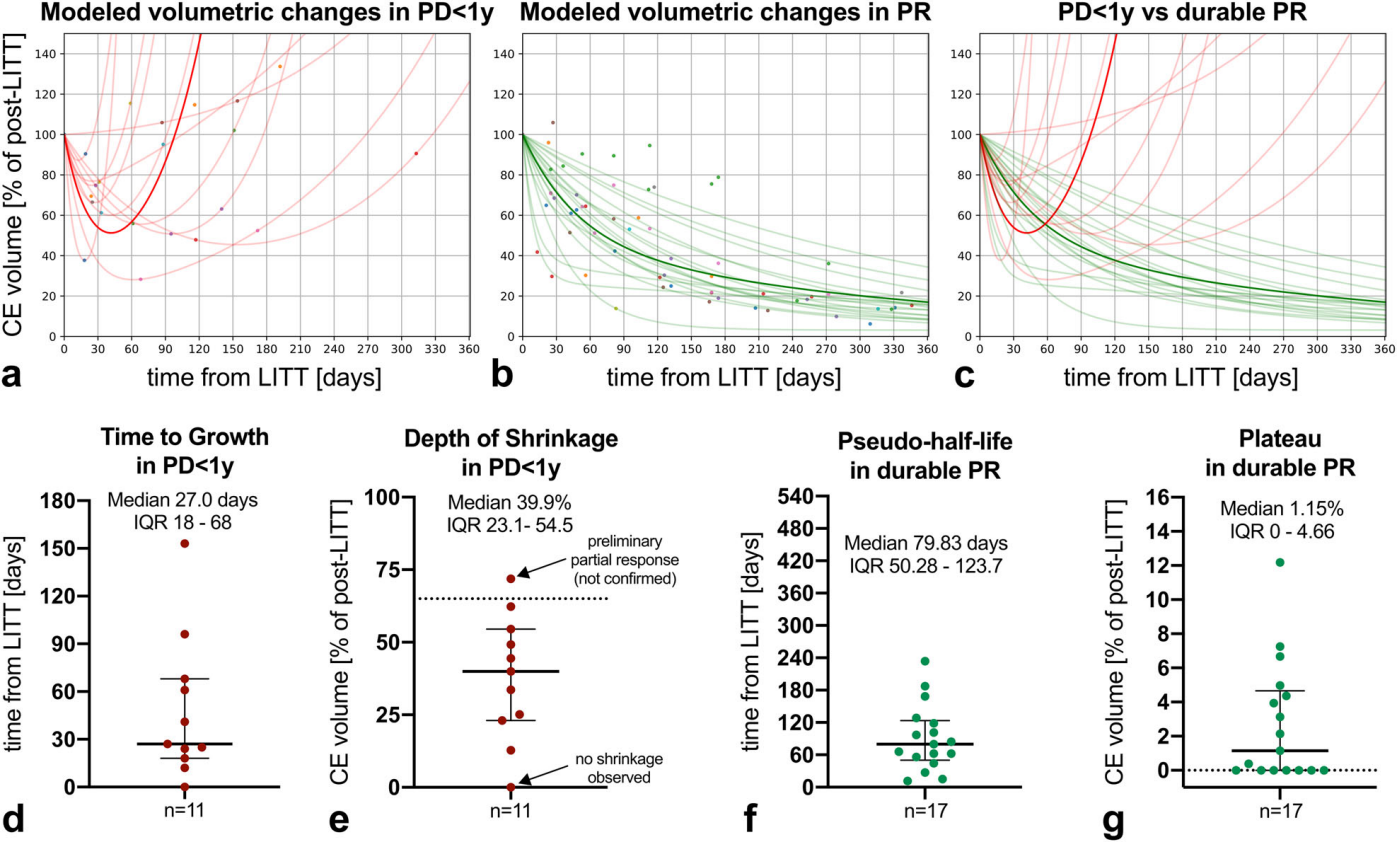
Mathematical modeling of volumetric changes following LITT. The observed values are shown as scatter dots with a lesion-specific color-coding (**a**, **b**). Lesion-specific fits are displayed as semi-transparent lines, and the high-opacity lines represent the overall trend of each group, obtained by plotting the median coefficients (**a**–**c**, where red lines correspond to PD < 1 y cases and green lines to durable PR cases). From the fitted models, it was possible to extract distributions of lesion-specific metrics describing the patterns of volumetric changes over time (**d**–**g**). One PD < 1 y case did not exhibit a post-LITT shrinkage, while another PD < 1 y case showed a pronounced post-LITT shrinkage (71.9%), meeting the criteria for preliminary partial response (**e**). CE, contrast-enhancing; IQR, interquartile range; PD < 1 y, progressive disease within 1 year after LITT; PR, durable response

Eleven PD < 1 y cases (91.7%) and seventeen PR cases (100%) had sufficient timepoints to be included in the modeling (Fig. 2, Supplementary Figs. 5, 6). The PD < 1 y bi-exponential model (Eq. 1) fitted the observed data with a median *R*^2^ = 1.0 (Fig. 2a). All PD < 1 y cases showed an initial shrinkage followed by a regrowth with the exception of one case which showed immediate regrowth without shrinkage (Fig. 2a). For each fitted line, the minimum fitted volume can be used to calculate the depth of shrinkage (Fig. 2e), and the corresponding time can be interpreted as the time to tumor growth (TTG, Fig. 2d). The median depth of shrinkage was 39.9% (IQR 23.1–54.5) and the median time to tumor growth was 27 days (IQR 18–68). TTG showed an inverse correlation with ΔADC (post-LITT compared to pre-LITT, ρ = −0.69, *p* < 0.05), and a trend toward a correlation with pre-LITT ADC (ρ = 0.56, *p* = 0.07). No significant correlations were observed between TTG and post-LITT ADC, pre-LITT CE volume, post-LITT CE volume, or %change in CE volume. In addition to the PD < 1 y cases, data from one LTDC case exhibiting a late PD (28 months) could also be successfully fitted with the same model (Supplementary Fig. 5). The PR bi-exponential model (Eq. 2) provided an excellent fit of the data overall (median *R*^2^ = 0.99 for PR, Fig. 2b), with the exceptions of two cases that showed an atypical shrinkage kinetic, with a decrease in volume at FU1, followed by a ~ 6-month stable phase, and then finally a marked progressive shrinkage (*R*^2^ = 0.94 and *R*^2^ = 0.81, respectively, Supplementary Fig. 6). The fitted values can be used to calculate a “pseudo-half-life” (Fig. 2f), corresponding to the time required for the lesion to become 50% of the post-LITT volume (different from a pure half-life of a mono-exponential fit). Finally, the fitted plateau term provides an estimate of the minimal residual CE volume in durable PR lesions (Fig. 2g). The median pseudo-half-life was 79.83 days (IQR 50.28–123.70), with a marked heterogeneity across lesions. The two lesions showing a ~ 6-month stable phase had a much longer pseudo-half-life (233.8 and 168.6 respectively), while 3 other lesions with a pronounced early-phase volume decay had a pseudo-half-life < 30 days (Supplementary Fig. 6). No significant correlations were observed between the pseudo-half-life and pre-LITT, post-LITT, or changes (post-LITT compared to pre-LITT) in ADC or CE volume.

The visualization of the fitted curves for both the PD < 1 y and the durable PR categories confirms that the CE volume shrinkage in the early follow-ups after LITT exhibits a high degree of overlap between categories (Fig. 2c). According to the fitted values for the two groups, the earliest pair of timepoints with high accuracy (≥ 0.8) in distinguishing PD < 1 y cases from PR cases are 23 days and 125 days after LITT (*p* < 0.01), with accuracy 0.82 (90% CI: 0.68–0.91), specificity 1.0 (0.86–1.0), sensitivity 0.55 (0.24–0.68). However, a more conventional pair of timepoints of 30 days and 90 days achieved a comparable diagnostic performance (*p* < 0.01, accuracy/specificity/sensitivity: 0.79/1.0/0.45).

### Diffusion changes following LITT

**Fig. 3:**
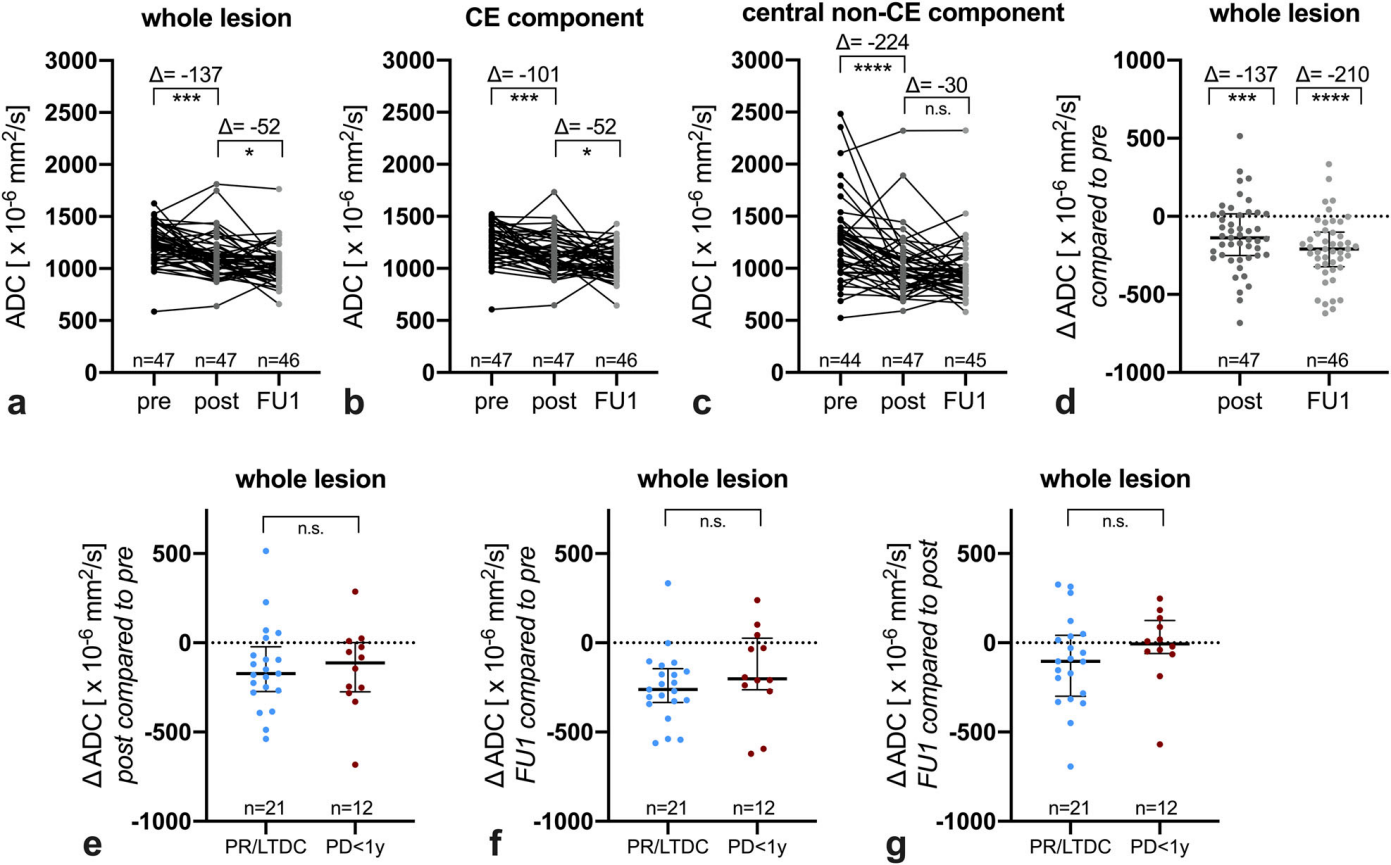
Diffusion changes following LITT. The whole lesion shows an ADC drop immediately after LITT, with a further ADC decrease at the following scan (**a**, **d**). The immediate pre-to-post-LITT ADC decrease in the whole lesion is primarily due to a marked ADC drop in the central non-CE area (**c**), while the ADC drop in the CE tissue is less pronounced (**b**). On the following scan, the further ADC decrease in the whole lesion is ascribable to diffusion changes in the CE component (**b**), while the ADC values of the central non-CE area are overall stable (**c**). The percent change of CE volumes at such timepoints is substantially similar between responding and progressing lesions (**e**–**g**). ADC, apparent diffusion coefficient; CE, contrast-enhancing; FU1, first follow-up scan; LTDC, long-term disease control; pre, pre-LITT scan; post, immediate post-LITT scan; PD < 1 y, progressive disease within 1 year after LITT; PR, durable response

The LITT procedure determined an ADC decrease of the whole lesion (–137 × 10^−^^6^ mm^2^/s median difference, *p* < 0.001, Fig. 3a, d), more prominent in the central non-CE component (−224 × 10^−^^6^ mm^2^/s median difference, *p* < 0.0001, Fig. 3c) and less pronounced in the CE component (−101 × 10^−^^6^ mm^2^/s median difference, *p* < 0.001, Fig. 3b). This overall trend was confirmed when plotting the data only from lesions imaged with similar DWI acquisition parameters (1.5 T, TE ranging 76–116 ms, TR ≥ 3000 ms, Supplementary Fig. 7) that should warrant maximal consistency in ADC measurements [32, 33]. The first post-LITT follow-up scan (FU1) showed a further milder ADC decrease (–52 × 10^−^^6^ mm^2^/s median difference in the whole lesion, *p* < 0.05, Fig. 3a, d). Missing observations due to the absence of DWI datasets (*n* = 1 on the FU1 timepoint, Fig. 3a–d) or due to the absence of a non-CE central component (*n* = 3 in the pre-LITT timepoint, and *n* = 1 in the FU1 timepoint, Fig. 3c) were excluded from the corresponding statistical analysis. Notably, there was a trend in the correlation between the degree of further ADC decrease and the time interval between the post-LITT and FU1 (ρ = 0.28, *p* = 0.058), suggesting that this additional dip in ADC values after LITT can be seen mainly in early follow-up scans. The magnitude of ADC decrease on the post-LITT and FU1 timepoints did not significantly differ between groups (PR/LTDC vs PD < 1 y, Fig. 3e–g). Post-LITT ADC changes were not significantly impacted by active systemic treatments nor extent of ablation category (Supplementary Fig. 8).

### Mathematical modeling of diffusion changes

**Fig. 4:**
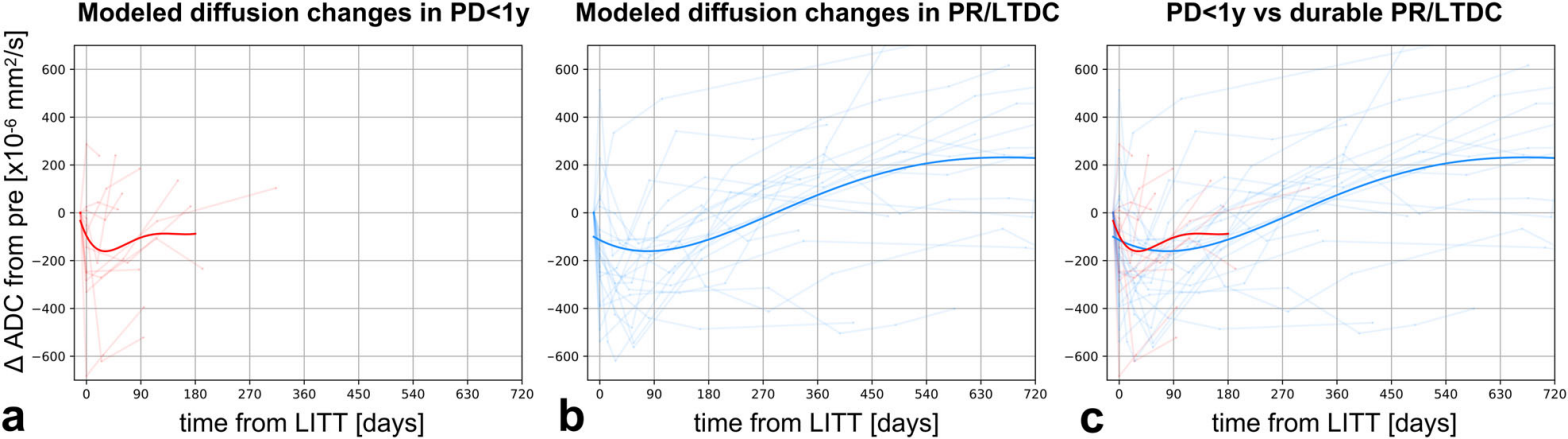
Mathematical modeling of diffusion changes following LITT. The observed values are shown as semi-transparent spider plots, while the overall fits are displayed as high-opacity lines. Red lines correspond to PD < 1 y cases (**a**, **c**) and blue lines to PR/LTDC cases (**b**, **c**). ΔADC, difference between ADC at a certain timepoint and the pre-LITT ADC; LTDC, long-term disease control; PD < 1 y, progressive disease within 1 year after LITT; PR, durable response

While ADC changes over time were heterogeneous across lesions, the proposed mathematical modeling allowed to visualize the overall trend in the response groups (Fig. 4, Supplementary Fig. 9). Both PR/LTDC and PD < 1 y showed an early ADC decrease after LITT, followed by a gradual ADC increase, which in PR/LTDC ultimately resulted in a higher ADC than baseline after ~ 6–9 months (Fig. 4, Supplementary Fig. 9).

## Discussion

**Fig. 5:**
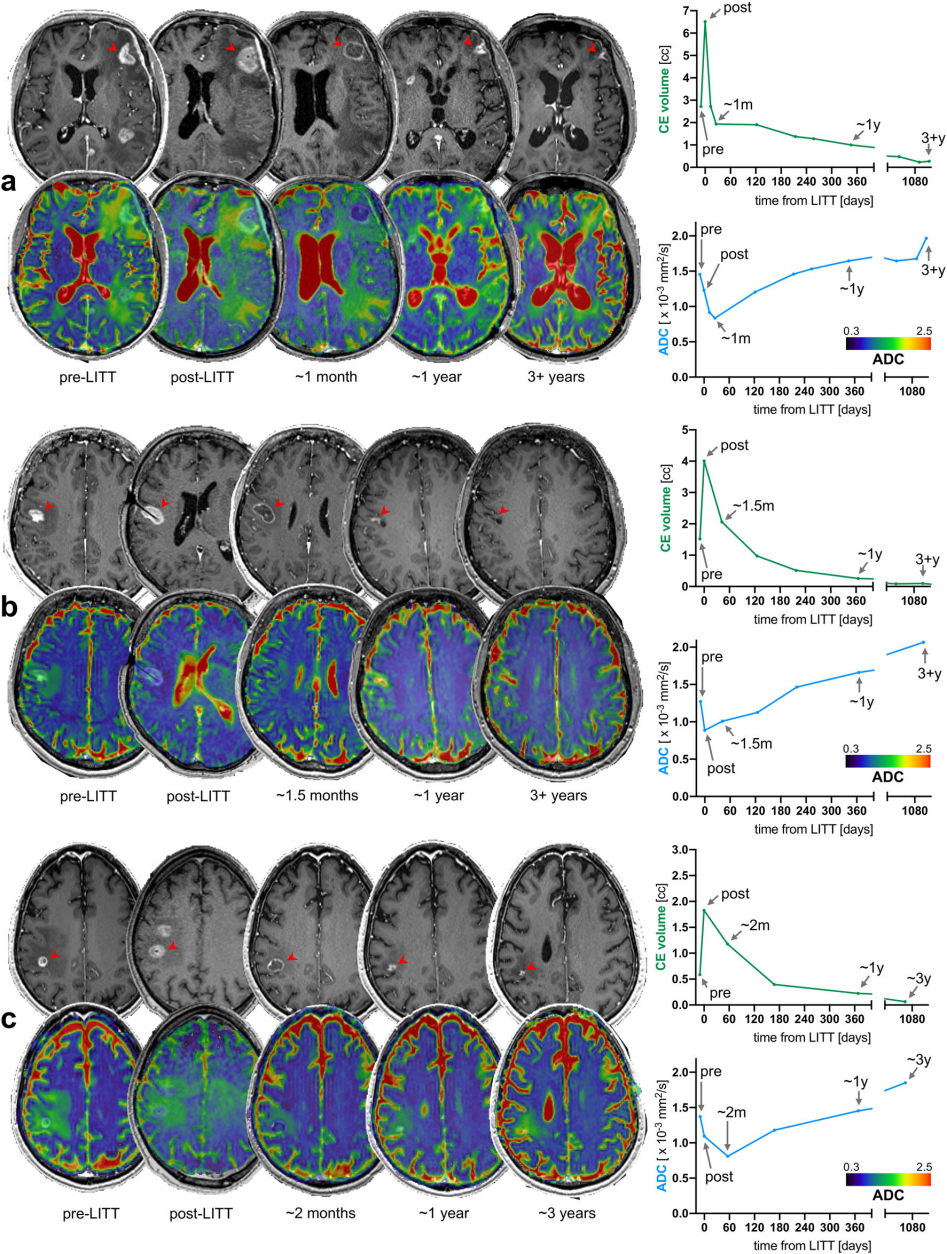
Typical radiographic pattern of durable response. Lesions exhibiting durable response (three representative cases in **a**–**c**) typically show an immediate post-LITT enlargement, especially of the CE component, associated with a decrease in ADC values, more pronounced in the central non-CE region. 1–2 months after LITT, the CE component shows a marked volume decrease, while the central non-CE region becomes larger, and ADC values can further decrease or remain stable. In the following months/years, the lesion shrinks more gradually until only some small residual CE tissue remains, while ADC values progressively increase above the pre-LITT values. ADC, apparent diffusion coefficient; CE, contrast-enhancing; pre, pre-LITT scan; post, immediate post-LITT scan

In this study, we characterize the radiographic changes of brain metastases receiving LITT and propose mathematical models to describe them. Considering the observed and modeled data, as well as case-by-case visual assessments, we propose a characterization of the typical radiographic pattern of lesions responding to this treatment (Fig. 5). Lesions showing durable response typically exhibit an immediate post-LITT enlargement, more pronounced in the CE component. Around 1–2 months after LITT, the central non-CE component further enlarges, while the CE component shrinks as part of a two-phase decay of the CE volume (a sharp early shrinkage followed by a more gradual late shrinkage) that results in a small residual CE area on long-term follow-up images. As for diffusion imaging, responding lesions typically exhibit a post-LITT ADC decrease and possibly a further ADC decrease 1–2 months after LITT, followed by a progressive ADC increase that rises above pre-LITT ADC on long-term follow-up images. These findings are overall consistent with the current understanding of LITT-induced modifications, as discussed further.

Laser ablation determines a lesion enlargement that has been previously described in other studies, although the mean/median extent of this enlargement is reportedly variable: ~ 77% in the present study (and ~ 133% in lesions showing response or long-term stability), ~ 278% in Rao et al [13], ~ 26% in Carpentier et al [11], ~ 45% in Beechar et al [12], ~ 64% in Torres-Reveron et al [7]. This volumetric increase is mainly linked to a heat-induced BBB permeability [29], and the variability in the reported values is likely due to some differences across studies, including differences in the timing of the post-LITT imaging (minutes or 24 h or even weeks after the procedure), technical differences in imaging acquisition and size assessment, and possibly technical differences in the LITT procedure. As for the volumetric response in the later follow-up scans, literature is rather sparse. In a seminal article, Schwabe et al proposed to fit a mono-exponential decay at the whole-cohort level to model the volumetric changes over time in LITT-treated lesions (*R*^2^ = 0.81), and the half-life of their exponential model can be calculated as ~ 38 days [21]. Our approach differs in two aspects, which explain the higher *R*^2^ scores in our study (median 0.99). First, we propose a bi-exponential fit to account for a two-phase decay (phase one: BBB permeability resolution; phase two: actual antitumoral effect) that is particularly evident in some lesions (Supplementary Fig. 6). Of note, the bi-exponential model provides a better fit for lesions showing a two-phase decay, while it does not worsen the fit of lesions that may show a one-phase decay since a bi-exponential model can approach a mono-exponential model in these cases (where phase one and phase two are similar in shrinkage rate, in which case the model simply converges to β_1_ ~ β_2_, and/or to α ~ 0 or α ~ 1). Second, we fit lesion-specific models (as opposed to a whole-cohort model) to account for response heterogeneity across lesions, as some lesions shrink much faster than other ones. In addition to providing better model fits, lesion-specific models allow to extract lesion-specific metrics (e.g., pseudo-half-life for responders, time to growth for progressors) that can potentially be used to classify lesions and as clinical trial radiographic outcomes, as demonstrated in other studies applying lesion-specific model fits [34]. Additionally, they allow to interpolate size values between timepoints for each lesion, which we used to evaluate the optimal scan times to maximize the accuracy to identify progressing lesions. In this analysis, we showed that follow-up scans at 23 days (~ 3 weeks) and 125 days (~ 4 months) may optimize this distinction (accuracy/specificity/sensitivity: 0.82/1.0/0.55), but a more conventional option of ~ 1-month and ~ 3-month follow-up scans is probably non-inferior (0.79/1.0/0.45). While the actual progression can be mostly seen at 3–4 months, it is important to acquire an earlier scan at 3–4 weeks to establish a nadir that serves for further detection of progression. As explained, this analysis is based on the model fits and may have limited value in the rare responding cases that do not follow a bi- or mono-exponential shrinkage (e.g., *n* = 2 lesions in our cohort showing size stability for ~ 6 months after LITT before shrinking more pronouncedly). Finally, other authors showed spider plots of size measurements over time [12, 13]. While model fits were not performed in these studies, the visual inspection of the spider plots suggests that responding lesions in these cohorts follow an exponential-like shrinkage, as well.

As for diffusion imaging, our observed ADC reduction after LITT is probably related to heat-induced coagulative necrosis, which was described in previous LITT articles, especially in the central region of the lesion [20–22, 25], and is known to appear as restricted diffusion on MRI [35, 36]. The following ADC increase in responding lesions, that rises above pre-LITT ADC in long-term follow-up scans, can be interpreted as a clearance of necrotic debris accompanied by a reduction in the density of tumor cells, which are possibly replaced by connective tissue (e.g., scar tissue), given that ADC is a proxy of cell density [37, 38]. While this can be considered the typical ADC pattern of responding lesions, it is important to note that other patterns can be seen. For instance, a subset of responding lesions exhibited increased post-LITT ADC values and/or an atypical ADC evolution on follow-up scans (Supplementary Fig. 10). A possible explanation for these atypical observations is that ADC measurements can be influenced by the presence of areas of liquefactive necrosis and, most importantly, of vasogenic edema, which can both be seen in LITT-treated areas [25, 39] and can both result in higher ADC values [40–43]. Therefore, the complex interplay between coagulative necrosis, liquefactive necrosis, and vasogenic edema explains the heterogeneity in ADC patterns across responding lesions.

In this study, early radiographic changes were not significantly different between responding and progressing lesions. A previous study suggested that a more marked post-LITT volumetric increase is seen in responding lesions [19]. While a larger lesion increase is potentially interpretable as a more radical ablation, we only saw a trend in this direction in this cohort, and we argue that the degree of post-LITT enlargement is only a rudimental surrogate of the extent of ablation. Notably, even a more refined method based on subtraction maps between post- and pre-LITT scans [2, 3] yielded an estimated extent of ablation that only showed a trend toward being more radical in responders (not a significant statistical association). Overall, these results confirm that the evaluation of the extent of ablation is a complex task, and current methodologies including the subtraction maps may achieve imprecise estimates, as previously discussed [2]. However, it is also possible that the present study is underpowered to confirm a significant association of treatment outcomes with post-LITT enlargement and with subtraction map-derived extent of ablation due to a relatively small sample size. Similarly, one study on glioblastoma patients reported that areas exhibiting a post-LITT ADC increase were more likely to show future recurrence [44]. It could be argued that an ADC decrease reflects a more prominent coagulative necrosis, suggesting LITT effectiveness. Our results suggest that this may be true only to some extent since the post-LITT ADC dip was associated with a longer time to growth in progressing lesions, but only showed a non-significant trend between groups with different outcomes. Overall, LITT-induced ADC changes appear to be complex and multifactorial, and not yet confirmed as a reliable marker of LITT success.

This study has some limitations, including its retrospective design and the relatively small sample size. Another limitation is the heterogeneity in the imaging protocols across timepoints due to follow-up imaging being acquired on different scanners with different techniques and pulse sequence parameters, which may yield some degree of inconsistency when comparing volumes and diffusion metrics over time. However, T_1_-post images were almost entirely acquired with BTIP-compliant high-resolution isometric voxel size, therefore the volumetric estimates of this study overall meet state-of-the-art standards. As for ADC measurements, inhomogeneity in acquisition factors may have introduced some degree of inaccuracy, especially when comparing time series with mixed sequences obtained at 1.5 T and 3.0 T. In this study, we did not apply dedicated normalization strategies to correct for this potential issue, but we confirmed that the post-LITT ADC drop was also appreciable in a selected subset of lesions with matching acquisition parameters. Another limitation is that it was not possible to study the association of post-LITT radiographic changes with previous RT exposure (due to the rarity of RT-naïve lesions, *n* = 4), nor with specific systemic treatment regimens (due to heterogeneity in combination schemes). In addition, the mathematical models have some assumptions, which dictate the data interpolation between timepoints. While the volumetric fits in the responders is arguably robust, given the goodness of the fit measured on many timepoints, the fits in the progressors group are more at risk of overfitting due to the few available timepoints. However, other studies validated bi-exponential fits to model tumor shrinkage and growth [34, 45]. More importantly, the results of our mathematical models should be considered as descriptive of the volumetric and diffusion patterns, rather than predictive. Finally, T_2_-weighted images and pre-contrast T_1_-weighted images were not acquired during the immediate post-LITT scan, which prevented the sub-segmentation of the classic 5 post-LITT regions [21].

## Conclusion

Brain metastases responding to LITT typically exhibit an early volume increase with diffusion restriction, followed by a bi-exponential volume shrinkage accompanied by a gradual diffusion increase. However, early volumetric and diffusion changes may not be reliable markers to predict LITT success, since they do not clearly differ between responding and progressing lesions. Follow-up scans may be performed at 3 weeks and 4 months (or at 1 and 3 months) to maximize the accuracy in identifying progressors.

## Supplementary information

ELECTRONIC SUPPLEMENTARY MATERIAL

## Abbreviations

ADC: Apparent diffusion coefficient
BBB: Blood-brain barrier
BM: Brain metastasis
CE: Contrast-enhancing
DWI: Diffusion-weighted imaging
FU1: First follow-up MRI after post-LITT MRI
IQR: Interquartile range
LITT: Laser interstitial thermal therapy
LTDC: Long-term disease control
MRI: Magnetic resonance imaging
PD < 1 y: Progressive disease within 1 year after LITT
PR: Partial response (durable)
RN: Radiation necrosis
RT: Radiation therapy
SD < 1 y: Stable disease and lost to follow-up within 1 year after LITT
SRS: Stereotactic radiosurgery
T_1_-post: Post-contrast T_1_-weighted images
TTG: Time to tumor growth
