# Supplementary material for: Volumetric and diffusion MRI longitudinal patterns in brain metastases after laser interstitial thermal therapy
Source: Eur Radiol. 2025 Apr 18;35(10):5981–93. doi: 10.1007/s00330-025-11587-0 (PMC12417287; doi:10.1007/s00330-025-11587-0)
Supplement: Supplementary file 1 — ELECTRONIC SUPPLEMENTARY MATERIAL [file 330_2025_11587_MOESM1_ESM.pdf]

Volumetric and diffusion MRI longitudinal patterns in brain metastases after laser interstitial thermal therapy

ELETTRONIC SUPPLEMENTAL MATERIAL

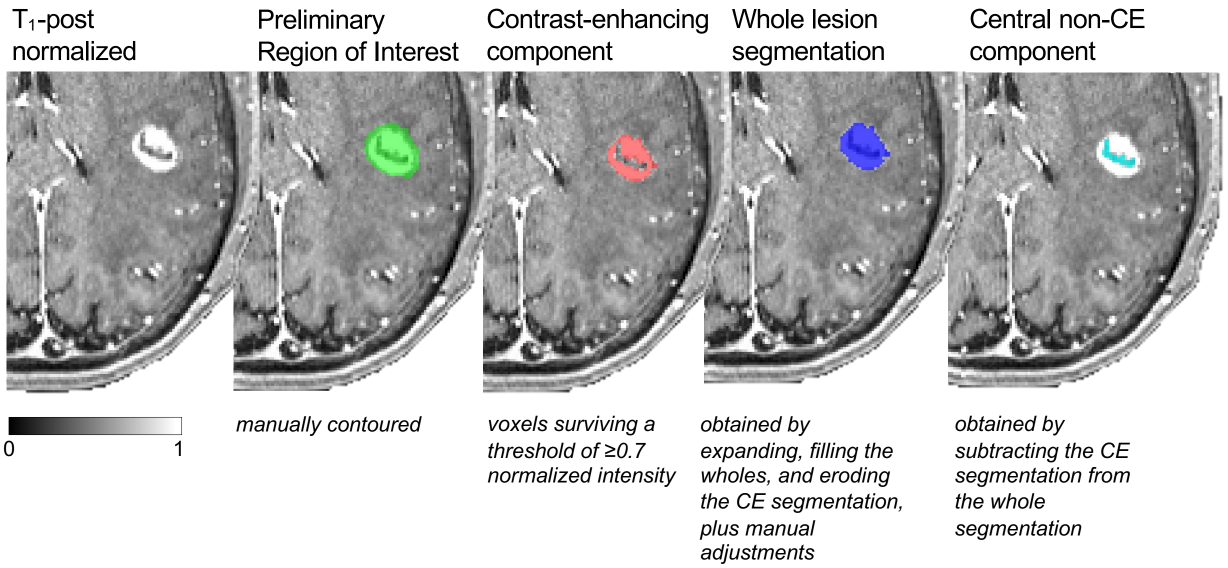

Supplementary Figure 1. Segmentation method. T_1-post images were processed using the *afni* built-in *3dUnifize* function and then divided by the maximum value within the image, therefore obtaining an intensity-normalized T_1-post, whose values ranged from 0 to 1 (left panel). The tumor region was manually contoured, to obtain a preliminary approximative region of interest (ROI) that contained the tumor (green). Voxels belonging to the preliminary ROI and surviving an intensity threshold of ≥0.7 on the normalized T_1-post image were included in the “contrast-enhancing (CE) segmentation” (red). A concatenated command with the *fsl* built-in functions *-dilM*, *-fillh*, and *-ero* were used to expand, fill the holes, and then erode the CE segmentation. The *-fillh* command fills the holes, to obtain a preliminary segmentation that includes both the CE tissue and the central non-CE component. Since *-fillh* only works if the holes to fill are surrounded by the CE segmentation in all three dimensions with no gaps, adding a preliminary command to expand to expand the CE segmentation by one voxel (*-dilM*) guarantees that the gaps are closed and the holes can be filled. The subsequent command to erode the segmentation (*-ero*) counteracts any undesired segmentation dilation on the external side of the lesion. This is an empirical method to obtain a preliminary approximative segmentation that includes both the CE tissue and the central non-CE component. The final “whole lesion” segmentation (blue) was obtained with minimal manual adjustments applied to this preliminary segmentation. Finally, the segmentation of the “central non-CE component” (light blue) was simply obtained with the subtraction between the “whole lesion” segmentation and the “CE segmentation”.

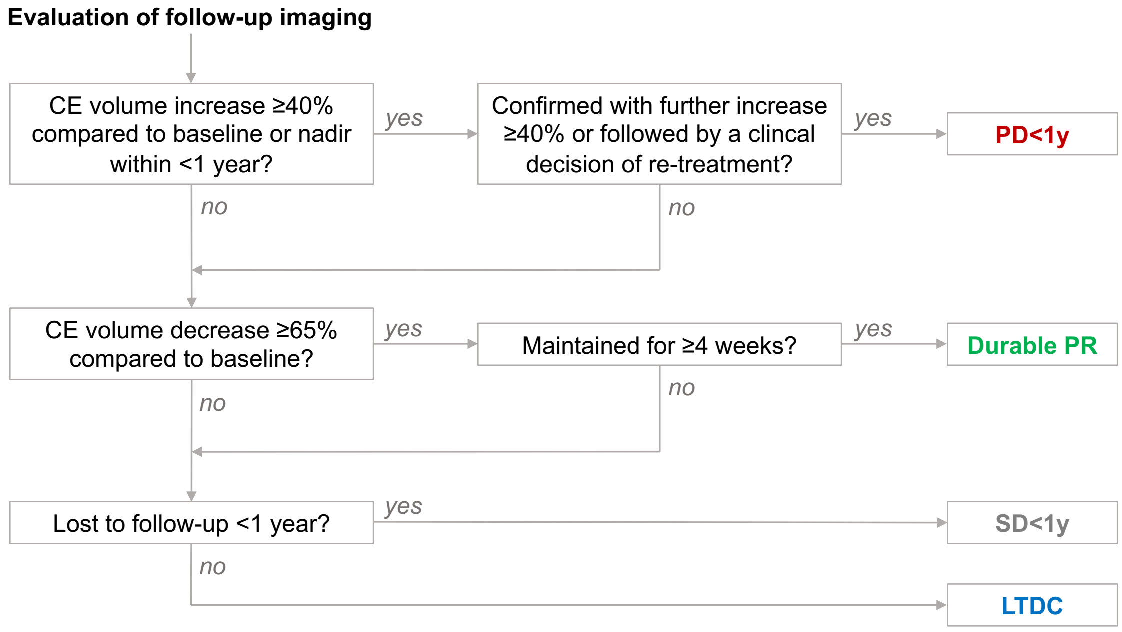

Supplementary Figure 2. Flow-chart showing the lesion categorization according to treatment response. The rationale behind this categorization is that both LTDC and PR can be considered a success of the LITT procedure, PD<1y can be considered an unsuccessful clinical result, while lesions SD<1y are relatively non-informative in terms of LITT success.
LTDC = long-term disease control (lesion showed stability at least for one year after LITT), PD<1y = progressive disease within 1 year after LITT, PR = durable response, SD<1y = stable disease before 1 year: lesions that appear stable at the time of censoring (i.e., when the patient was lost to follow-up) and for which censoring happened before 1 year after LITT.

Supplementary Methods
(Continuation of "Methods: Mathematical modeling of radiographic changes").

Analysis to evaluate the optimal timepoints for follow-up scans, according to the volumetric modeling.
The bi-exponential volumetric modeling was performed at the single-lesion level for the PD<1y group and the durable PR group. Assuming that the model fits the data reliably (which is supported by the visualization of the model fits and by the R^2 results), the fitted curves provide modeled interpolated volumes for each theoretical day of follow-up. We wrote a code that loops through any *pair* of days within the first year of follow-up ("day 1" and "day 2"), and calculates the percent change between the two. If the percent change exceeds +40%, the lesion is categorized as "progression", otherwise as "non-progression". For each pair of days, a contingency table was built to compare the expected categorizations "progression" and "non-progression" vs the ground-truth categorizations PD<1y and durable PR, a *p*-value was calculated with a Fisher's exact test, and accuracy/sensitivity/specificity of the test to predict PD<1y as "progression" was calculated. The suggested optimal timepoints were the *earliest* pair of timepoints that achieved an accuracy ≥0.8, with accuracy = (true positives + true negatives) / (all).

Supplementary Table 1. Voxel dimensions of post-contrast T_1-weighted images

Timepoint	Voxel dimensions: x dimension × y dimension × z dimension (n of lesions)
Pre-LITT	n=44 imaged with voxel size ≤1 × ≤1 × ≤1 mm, *isotropic*<br>n=2 imaged with voxel size ≤1 × ≤1 × ≤1.5 mm, *non-isotropic*<br>n=1 imaged with voxel size 0.23 × 0.23 × 3 mm, *non-isotropic*
Post-LITT *	n=46 imaged with voxel size ≤1 × ≤1 × ≤1 mm, *isotropic*<br>n=1 imaged with voxel size 0.63 × 0.63 × 1 mm, *non-isotropic*
FU1 after LITT	n=39 imaged with voxel size ≤1 × ≤1 × ≤1 mm, *isotropic*<br>n=6 imaged with voxel size ≤1 × ≤1 × ≤1.5 mm, *non-isotropic*<br>n=1 imaged with voxel size 0.5 × 0.5 × 3 mm, *non-isotropic*<br>n=1 imaged with voxel size 0.45 × 0.45 × 5 mm, *non-isotropic*
Other FU scans ¶	n=142 imaged with voxel size ≤1 × ≤1 × ≤1 mm, *isotropic*<br>n=9 imaged with voxel size ≤1 × ≤1 × ≤1.5 mm, *non-isotropic*<br>n=2 imaged with voxel size ≤1 × ≤1 × ≤3 mm, *non-isotropic*<br>n=2 imaged with voxel size ≤0.7 × ≤0.7 × ≤6 mm, *non-isotropic*

* Post-LITT scans were obtained immediately after the procedure, using a surface coil.
¶ This row groups all follow-up scans after the first follow-up (FU1) for each lesion, therefore including multiple scans for each lesion.

Supplementary Table 2. Pulse sequence parameters of diffusion-weighted imaging

Timepoint	Field strength (n of lesions)	Echo time (TE) (median and IQR)	Repetition time (TR) (median and IQR)
Pre-LITT	n=13 at 1.5 T<br>n=34 at 3.0 T	100 ms<br>(89–108)	10000 ms<br>(7000–10000)
Post-LITT *	n=47 at 1.5 T	96 ms<br>(96–96)	4500 ms<br>(4500–5200)
FU1 after LITT	n=19 at 1.5 T<br>n=27 at 3.0 T	96.45 ms<br>(78–108)	8300 ms<br>(6800–10000)
Other FU scans ¶	n=42 at 1.5 T<br>n=96 at 3.0 T	96 ms<br>(91.5–108)	8700 ms<br>(7600–10000)

* Post-LITT scans were obtained immediately after the procedure, using a surface coil.
¶ This row groups all follow-up scans after the first follow-up (FU1) for each lesion, therefore including multiple scans for each lesion.

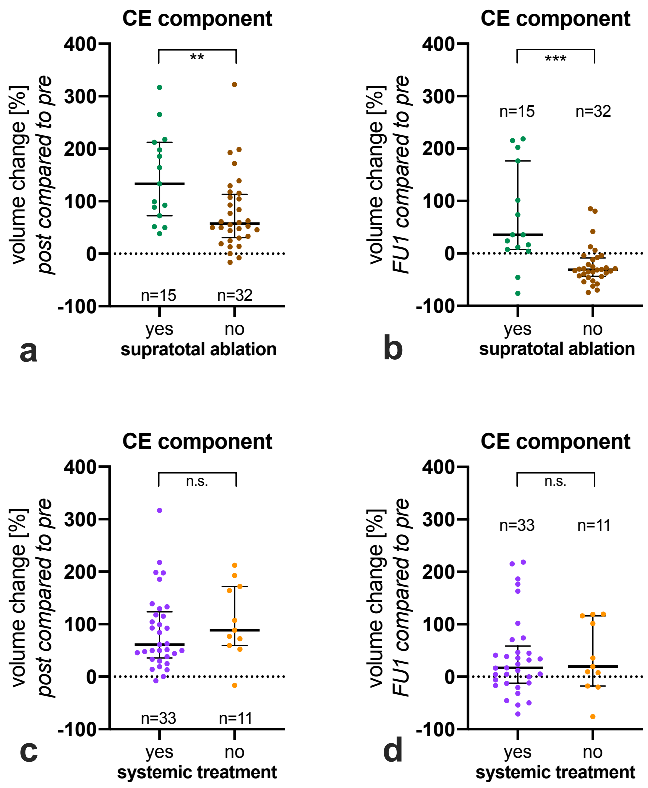

Supplementary Figure 3. Volumetric changes following LITT. Lesions with a “supratotal ablation” (estimated with the subtraction map approach) had a greater enlargement of the CE lesion component at post-LITT and FU1 timepoints, compared to lesions with an estimated “non-supratotal ablation”. No significant differences in the degree of enlargement of the CE lesion component were seen between lesions treated with systemic therapy and untreated lesions.

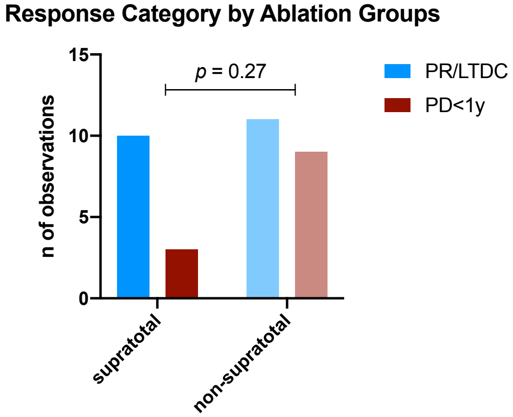

Supplementary Figure 4. A relatively higher fraction of responders was in the group with an estimated “supratotal ablation”, but not significant association was seen between the estimated extent of ablation and treatment outcomes.

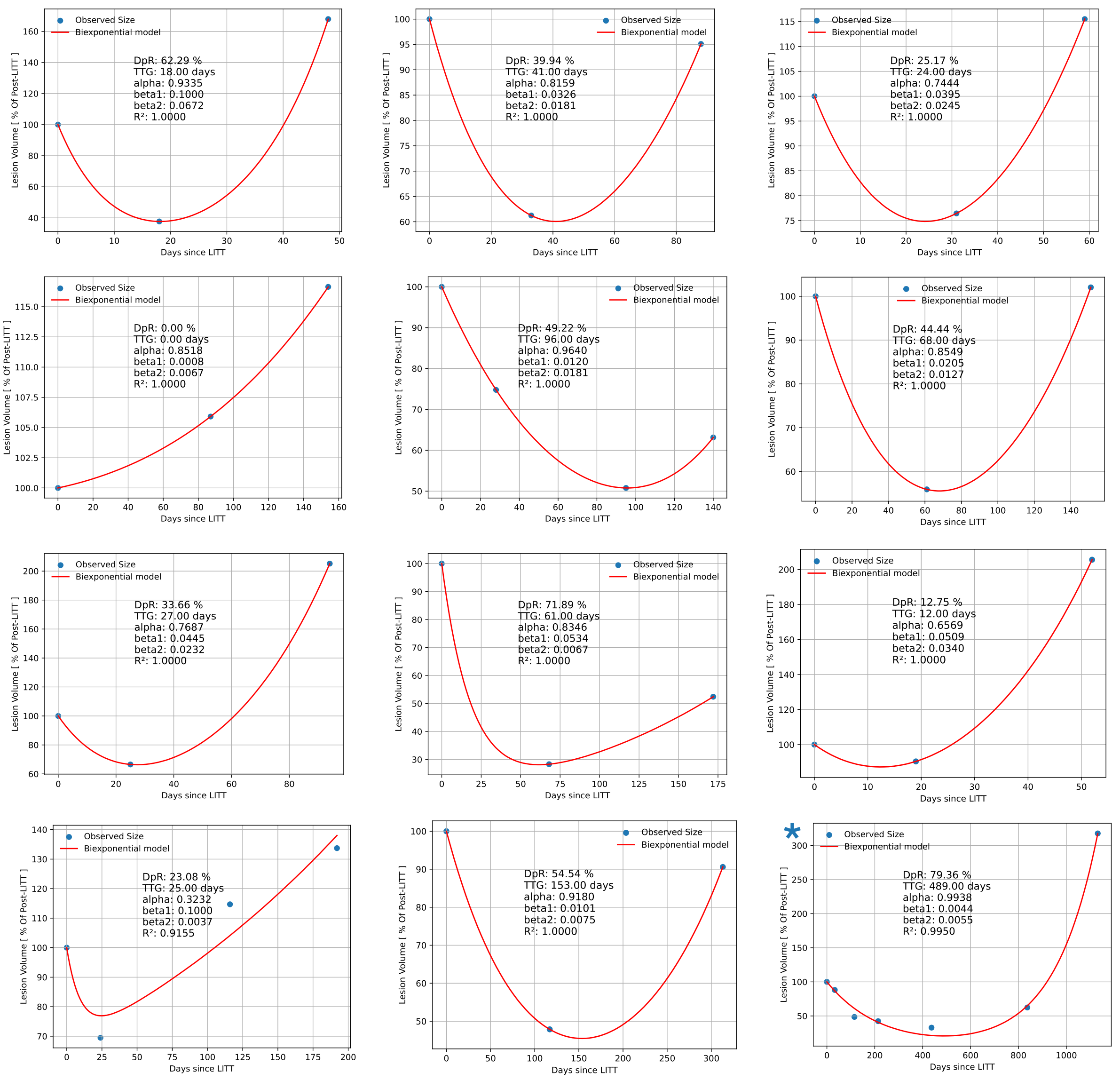

Supplementary Figure 5. Individual model fits of volumetric changes following LITT in the PD group. The biexponential fits (combined exponential shrinkage and exponential growth) are shown for each of the 11 lesions belonging to the PD<1y group, separately. Of note, this model successfully fitted one LTDC case that progressed at 28 months (asterisk). DpR = depth of response (or depth of shrinkage), TTG = time to growth.

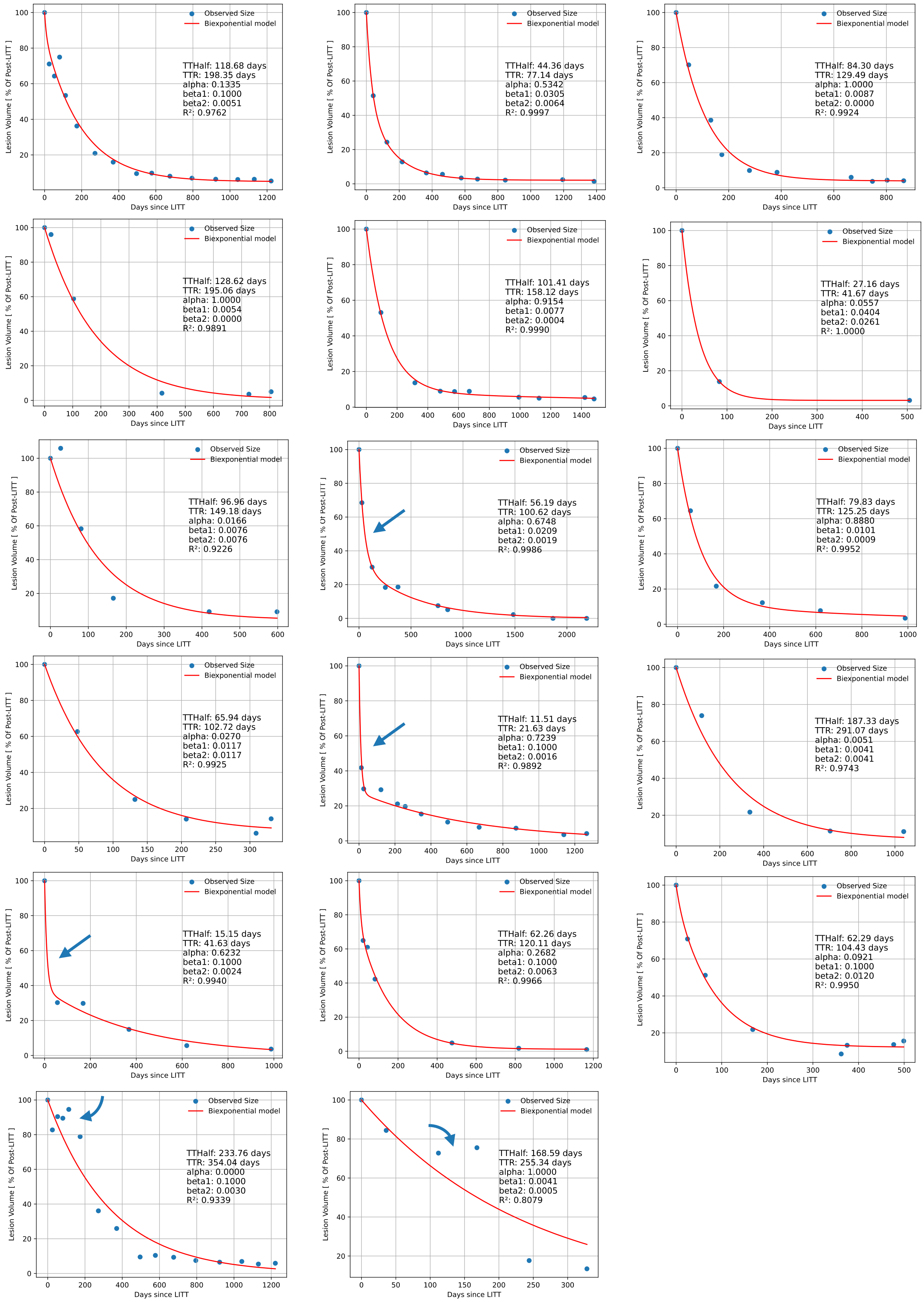
Observed Size
Biexponential model
Lesion Volume [% Of Post-LITT]
Days since LITT
TTHalf: 118.68 days
TTR: 198.35 days
alpha: 0.1335
beta1: 0.1000
beta2: 0.0051
R²: 0.9762
TTHalf: 44.36 days
TTR: 77.14 days
alpha: 0.5342
beta1: 0.0305
beta2: 0.0064
R²: 0.9997
TTHalf: 84.30 days
TTR: 129.49 days
alpha: 1.0000
beta1: 0.0087
beta2: 0.0000
R²: 0.9924
TTHalf: 128.62 days
TTR: 195.06 days
alpha: 1.0000
beta1: 0.0054
beta2: 0.0000
R²: 0.9891
TTHalf: 101.41 days
TTR: 158.12 days
alpha: 0.9154
beta1: 0.0077
beta2: 0.0004
R²: 0.9990
TTHalf: 27.16 days
TTR: 41.67 days
alpha: 0.0557
beta1: 0.0404
beta2: 0.0261
R²: 1.0000
TTHalf: 96.96 days
TTR: 149.18 days
alpha: 0.0166
beta1: 0.0076
beta2: 0.0076
R²: 0.9226
TTHalf: 56.19 days
TTR: 100.62 days
alpha: 0.6748
beta1: 0.0209
beta2: 0.0019
R²: 0.9986
TTHalf: 79.83 days
TTR: 125.25 days
alpha: 0.8880
beta1: 0.0101
beta2: 0.0009
R²: 0.9952
TTHalf: 65.94 days
TTR: 102.72 days
alpha: 0.0270
beta1: 0.0117
beta2: 0.0117
R²: 0.9925
TTHalf: 11.51 days
TTR: 21.63 days
alpha: 0.7239
beta1: 0.1000
beta2: 0.0016
R²: 0.9892
TTHalf: 187.33 days
TTR: 291.07 days
alpha: 0.0051
beta1: 0.0041
beta2: 0.0041
R²: 0.9743
TTHalf: 15.15 days
TTR: 41.63 days
alpha: 0.6232
beta1: 0.1000
beta2: 0.0024
R²: 0.9940
TTHalf: 62.26 days
TTR: 120.11 days
alpha: 0.2682
beta1: 0.1000
beta2: 0.0063
R²: 0.9966
TTHalf: 62.29 days
TTR: 104.43 days
alpha: 0.0921
beta1: 0.1000
beta2: 0.0120
R²: 0.9950
TTHalf: 233.76 days
TTR: 354.04 days
alpha: 0.0000
beta1: 0.1000
beta2: 0.0030
R²: 0.9339
TTHalf: 168.59 days
TTR: 255.34 days
alpha: 1.0000
beta1: 0.0041
beta2: 0.0005
R²: 0.8079

Supplementary Figure 6. Individual model fits of volumetric changes following LITT in the durable PR group. The biexponential fits (two-phase exponential decay with plateau) are shown for each of the 17 lesions belonging to the durable PR group, separately. For some lesions, a sharper early shrinkage was particularly evident (straight arrows). The model fitted the observed values satisfactorily, with the exception of two lesions showing a volume stabilization in the first ~6 months (curved arrows), before exhibiting a pronounced shrinkage. TTHalf = time to half (or pseudo-half-life), TTR = time to response.

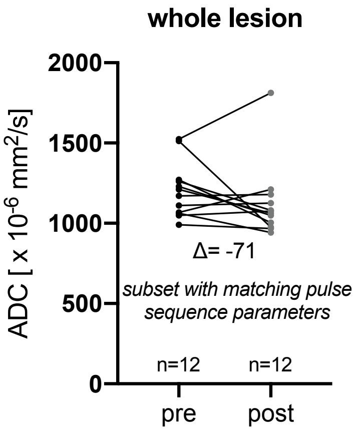

Supplementary Figure 7. Plot of ADC changes in lesions imaged with matching acquisition parameters. The plot shows the trend of decreasing ADC post-LITT in selected lesions scanned at 1.5 T, with TE ranging 76–116 ms, and with TR ≥3000 ms at *both* pre- and post-LITT timepoints. These ranges of acquisition parameters were chosen after consulting previous articles suggesting that some degree of inconsistency in ADC measurements can be seen between different field strengths, or with TE differences exceeding ~40 ms, or with TR differences if TR <3000 ms [1, 2].

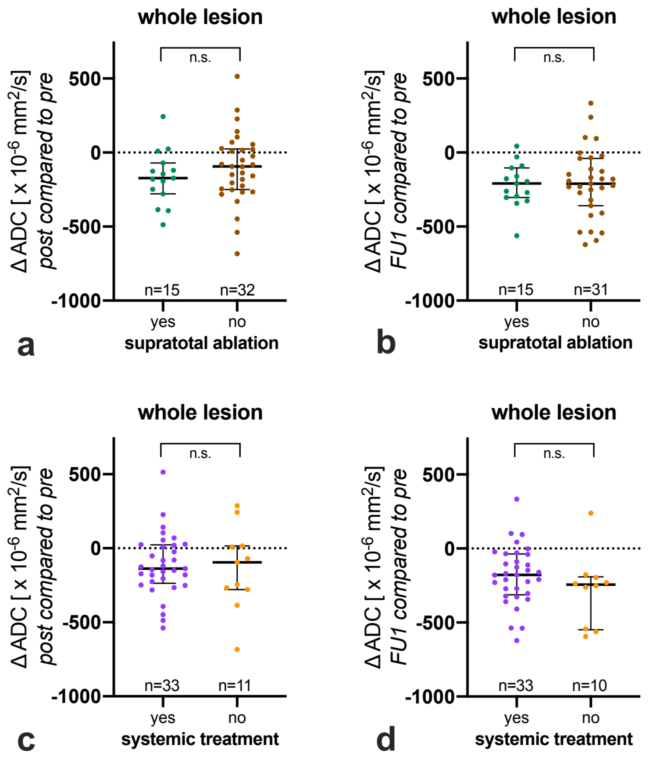

Supplementary Figure 8. Diffusion changes following LITT. No significant differences in the degree of ADC changes were seen between lesions with "supratotal" and "non-supratotal" ablations, nor between lesions treated with systemic therapies and untreated lesions.

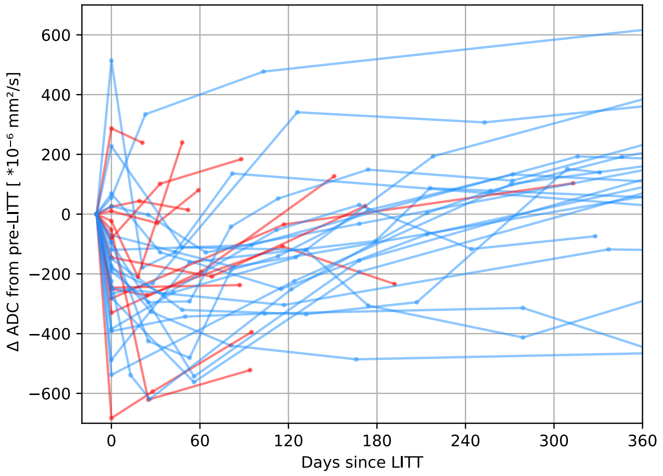

Supplementary Figure 9. Spider plots of diffusion changes following LITT. ADC values showed an overall decrease after LITT and then a subsequent progressive increase, in both the PD<1y group (red) and the LTDC/PR group (blue). Of note, some lesions showed an atypical behavior, with post-LITT ADC increase or with prolonged ADC reduction.

ΔADC = difference between ADC at a certain timepoint and the pre-LITT ADC, LTDC = long-term disease control, PD<1y = progressive disease within 1 year after LITT, PR = durable response.

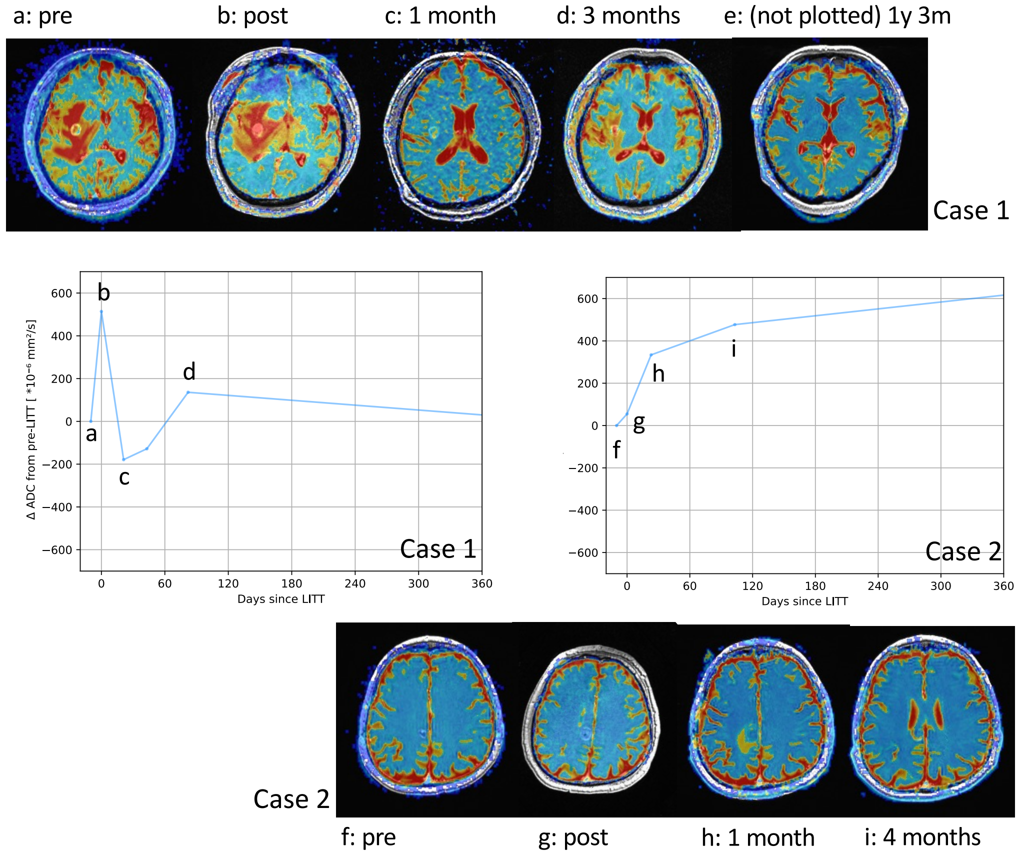

Supplementary Figure 10. Atypical diffusion MRI patterns after LITT success. The figure depicts two atypical cases of ADC increase after LITT in long-term responders with durable PR (case 1: the left plot and panels a–e; case 2: right plot and panels f–i). ADC color maps are co-registered and overlayed on T_1-post. Case 1 showed a prominent early post-LITT ADC increase (b), followed by an ADC drop at FU1 (c) and then a stabilization of ADC measurements (d). Case 2 showed a mild ADC increase post-LITT (g), that became gradually more marked in later follow-ups (h–i). “Atypical” increased ADC values in early post-LITT scans are possibly due to prominent vasogenic edema and/or liquefactive necrosis, that may prevail compared to coagulative necrosis. On late follow-ups after LITT, high ADC is seen also in typical cases and may be related to gliosis.

References:

1. Huisman TAGM, Loenneker T, Barta G, et al (2006) Quantitative diffusion tensor MR imaging of the brain: field strength related variance of apparent diffusion coefficient (ADC) and fractional anisotropy (FA) scalars. Eur Radiol 16:1651–1658. https://doi.org/10.1007/s00330-006-0175-8
2. Celik A (2016) Effect of imaging parameters on the accuracy of apparent diffusion coefficient and optimization strategies. Diagn. Interv. Radiol. 22:101–107
